# Equivalent magnetic circuit method of estimating iron losses in induction motor spindles

**DOI:** 10.1038/s41598-022-13055-x

**Published:** 2022-06-09

**Authors:** Lang Lü, Wanli Xiong, Can Hu

**Affiliations:** 1 College of Mechanical Engineering, Hunan Institute of Engineering, Xiangtan, 411104 China; 2 National Engineering Research Center for High Efficiency Grinding, Hunan University, Changsha, 410082 China; 3 School of Mechanical Engineering, Hunan University of Science and Technology, Xiangtan, 411201 China

**Keywords:** Engineering, Electrical and electronic engineering, Mechanical engineering

## Abstract

The iron losses in the motor of motorized spindles have a significant effect on their heat generation, thermal deformation, and machining accuracy. The equivalent magnetic circuit (EMC) method for estimating iron losses in the spindle motor is proposed, where the magnetic flux density distribution of any cross section inside the spindle motor is assumed as a uniform one. A mechanical loss separation method of no load running combined with a sudden loss of power supply is also proposed. The EMC method is verified by prototype experiment and a different analysis method comparison. The EMC does not need to solve complex electromagnetic fields, and to do 2D or 3D eddy current analysis and the corresponding post-processing. There is only need to perform a simple magnetic circuit calculation. Therefore, it can realize a fast analysis and prediction. The proposed mechanical loss separation method requires only one prototype during a whole testing process. There is no need for any other same prototype and a coupling device. It is simpler, and can eliminate the braking torque and electromagnetic losses of the spindle motor.

## Introduction

Motor or motorized spindles belong to a new kind of machine tool spindles, where middle transmission chains like belts or gears are cancelled, namely achieving no middle transmission chain or direct drive. They have prominent advantages over traditional mechanical spindles, e.g., compact structure, small inertia, fast dynamic response, high-speed (high productivity), high precision, wide speed range, low vibration and noise, and easy to realize automatic and precise control^1–4^. Motorized spindles have been increasingly applied to high-speed precision machining such as high-speed milling, grinding, and turning^5–7^.

As a drive motor of motorized spindles, induction motors (IMs) are always fed by a voltage source inverter. The output voltage waveform of the inverter is not a sinusoidal wave but a series of rectangular pulse waves^8,9^. Numerous harmonic components from inverter supply are introduced into the stator winding current of the spindle motor. This makes the iron losses of the spindle motor in their physical origins more complicated. It is a challenge to analyze and predict them accurately. The calculation results of motorized spindles such as heat generation, thermal deformation^10,11^, and rotating error are further affected when they are developed and designed. Therefore, to find a fast and accurate prediction method of iron losses in the spindle motor is significant for reducing the development and design cost of motorized spindles, and improving their performance.

The ongoing study of iron losses in motors has been done due to their complex generation mechanisms^12–14^. The proper modeling of soft magnetic materials (SMMs) is the basis of performing the exact analysis and prediction of iron losses in motors. There are two basic types of loss models: frequency domain models and time domain models.

Various frequency domain models have been developed. Boglietti et al.^15,16^ presented a general loss model. The model is derived from the fact that any loss component contribution in it depends on the characteristics of supply voltage, and the Bertotti’s three-term loss separation model^17^ and the Steinmetz’s equation^18^. The Boglietti’s model is suitable for any voltage waveform supply. On the basis of the Boglietti’s model, variable parameter models^19,20^ were investigated for obtaining more accurate results over wide frequency and magnetic flux density ranges. In these models, the hysteresis and eddy current loss coefficients are assumed as the functions of the magnitude and frequency of the magnetic flux density. However, they need more experiment data to extract the model parameters before use.

Different time domain models have also been developed. Reinert et al.^21^ investigated a modified Steinmetz’s equation (MSE) in time domain to estimate the loss in SMMs under arbitrary flux waveforms. In time domain, Barbisio et al.^22^ developed a general loss model of SMMs with regard for minor hysteresis loops. However, the modeling^21,22^ is to model the magnetic characteristics of SMMs in the case of only an alternating magnetic field.

In order to describe the hysteresis phenomena and magnetic properties of SMMs under both alternating and rotating fields, a generalized vector hysteresis model^23,24^ in time domain was established from a traditional Chua-type vector hysteresis model^25^. A comparison study between frequency and time domain models was made^26^.

Time domain models^21–25^ can gain better accuracy than frequency domain models^15,16,19,20^ because of considering more realistic situations including magnetic saturation, hysteresis properties, magnetic field rotating and alternating, and distorted magnetic flux waveforms. However, they require extensively experimental tests to construct the database of the model parameters. In addition, they are always used along with the finite element. This slows down the calculation efficiency of the model.

The combination of the finite element analysis (FEA) and the loss models of SMMs has become a fashionable method of calculating iron losses in motors^8,27–31^. The FEA is used along with the Bertotti’s model to estimate iron losses in a slot-less permanent magnet synchronous motor (PMSM)^8^. The motor stator cores are respectively made from two different types of SMMs for a comparative study. The FEA is used together with the MSE to calculate the hysteresis loss in a surface-mounted PMSM^29^. The desired or accurate results can be obtained from the FEA, but it requires long enough time for the preparation work such as motor modelling, model meshing, and material definition. Also the computation is time-consuming. The analytical method based on Maxwell’s equations was studied to improve computational efficiency^32,33^. However, both FEA and analytical methods based on Maxwell’s equations are too complicated to be suitable for engineering designers.

In this paper, the equivalent magnetic circuit (EMC) method of estimating iron losses in the spindle motor is proposed, where the magnetic flux density distribution of any cross section inside the spindle motor is assumed as a uniform one. In the EMC, the problem of solving a complex electromagnetic field can be turned into a simple magnetic circuit calculation by the assumption. There is no need to solve a complex electromagnetic field, and to do 2D or 3D eddy current analysis and the corresponding post-processing. It needs to only perform a simple magnetic circuit calculation. It can realize a fast analysis and prediction, but its calculation accuracy is affected. A mechanical loss separation method of no load running combined with a sudden loss of power supply is also proposed to eliminate the braking torque and electromagnetic losses of the spindle motor. The proposed method is simpler. It requires only one prototype during a whole testing process. There is no need for any other same prototype and a coupling device. The EMC method is verified by prototype experiment and a different analysis method comparison.

## Equivalent magnetic circuit combined with loss model

### Equivalent magnetic circuit

**Figure 1:**
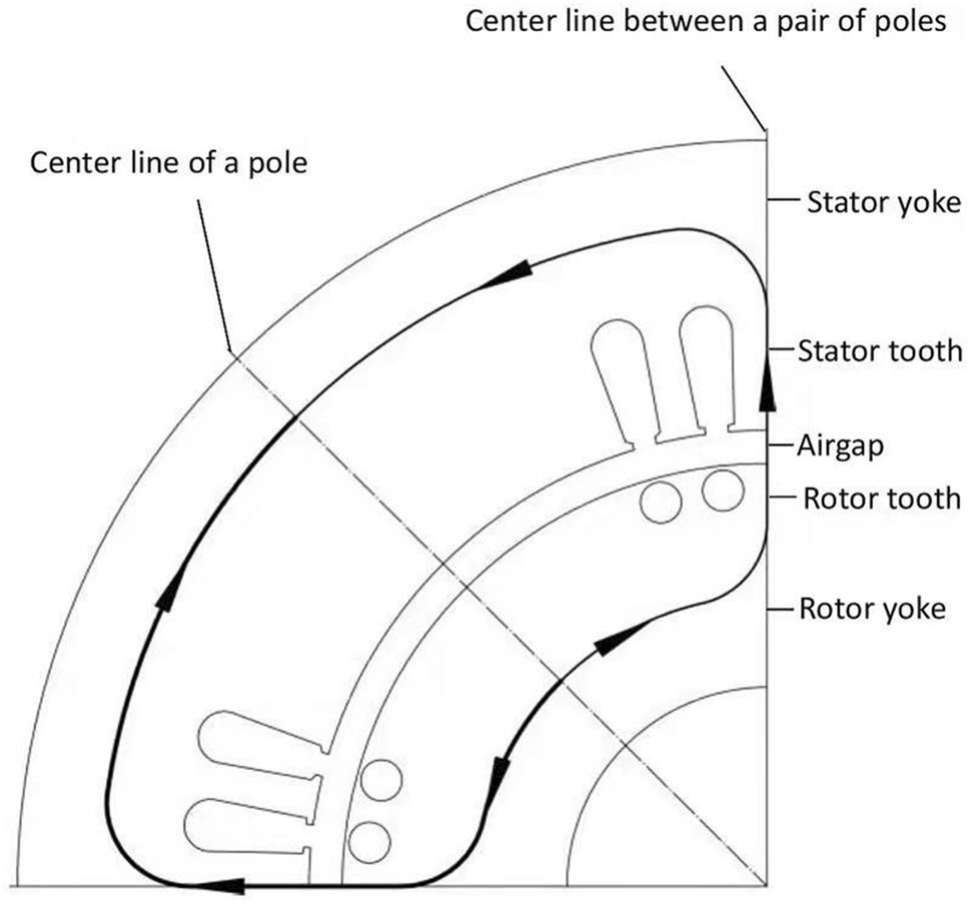
Typical magnetic circuit of IMs per pole.

The magnetic flux density distribution of any cross section inside motors is assumed as a uniform one. Based this assumption, the problem of solving a complex electromagnetic field can be simplified into a simple magnetic circuit calculation. A typical magnetic circuit of induction motors (IMs) per pole is shown in Fig. 1.

According to Ampere’s loop law, the integral result is independent of the path. A magnetic circuit which passes through the center line between two adjacent or a pair of magnetic poles, is usually chosen as one for calculation, as illustrated in Fig. 1. The magnetic circuit consists of five parts: airgap, stator tooth, stator yoke, rotor tooth, and rotor yoke segments. Only one half of the magnetic circuit is calculated because of its symmetry.

The magnetic flux of IMs per pole can be calculated from the following formula^34^

1 $$\Phi = \frac{E}{{2.22fN_{\phi } K_{{{\text{dp}}}} }},$$

where $E$ is an induced winding back electromotive force per phase, $N_{\upphi }$ denotes the number of stator winding series conductors per phase, and $K_{{{\text{dp}}}}$ is a stator winding coefficient which depends on the number of slots per pole per phase, the electrical angular degree between two adjacent slots, and the number of slots spanned by two effective sides of a winding coil.

The peak value of the magnetic flux density in airgap can be obtained from a mathematical expression as

2 $$B_{{\delta \text{,p}}} = F_{\text{s}} \frac{\Phi }{{\tau l_{{\text{ef}}} }},$$

where $F_{{\text{s}}}$ is the waveform amplitude coefficient of the magnetic flux density in airgap (the ratio of the peak value of the magnetic flux density in airgap to its average value) and it is a function of magnetic saturation occurring in stator and rotor core teeth, $\tau$ is the pole pitch expressed in that the circumference of a circular inner hole of the stator core is divided by the number of poles, and $l_{{{\text{ef}}}}$ denotes an effective core length.

The respective calculation formulas of the magnetic flux densities of stator and rotor teeth can be expressed as

3 $$B_{{\text{st,p}}} = F_{\text{s}} \frac{\Phi }{{A_{{\text{st}}} }},$$

4 $$B_{{\text{rt,p}}} = F_{\text{s}} \frac{\Phi }{{A_{{\text{rt}}} }},$$

where $B_{{\text{st,p}}}$ and $B_{{\text{rt,p}}}$ are the respective peak values of the magnetic flux densities of stator and rotor teeth, $A_{{{\text{st}}}}$ and $A_{{{\text{rt}}}}$ represent the respective sums of the flux area of every stator and rotor tooth per pole,$A_{{{\text{st}}}} = b_{{{\text{st}}}} Z_{1} /\left( {2p} \right)l_{{\text{s}}}$ and $A_{{{\text{rt}}}} = b_{{{\text{rt}}}} Z_{1} /\left( {2p} \right)l_{{\text{r}}}$. Where $b_{{{\text{st}}}}$ and $b_{{{\text{rt}}}}$ are the respective widths of stator and rotor teeth, $Z_{\text{1}}$ and $Z_{2}$ are the respective numbers of stator and rotor teeth or slots, $p$ is the number of pole pairs, $l_{\text{s}}$ and $l_{\text{r}}$ denote the respective net lengths of the laminated stator and rotor cores, $l_{\text{s}} \,=\,K_{{\text{Fe}}} l_{\text{1}}$ and $l_{\text{r}} \,=\,K_{{\text{Fe}}} l_{\text{2}}$.Where $K_{{\text{Fe}}}$ is a laminated coefficient, $l_{\text{1}}$ and $l_{\text{2}}$ are the respective lengths of the laminated stator and rotor cores.

Whether a stator yoke or a rotor yoke, their magnetic flux densities vary at different cross sections along the magnetic flux direction. When a yoke cross section goes through the center line between two adjacent poles, the magnetic flux density of the section reaches its maximum value. However, when a yoke cross section goes through the center line of a pole, the magnetic flux density of the section is exactly equal to zero, as shown in Fig. 1. Thus, the total magnetic flux of a yoke part is only one half of the magnetic flux per pole. The maximum magnetic flux densities of stator and rotor yoke parts can be formulated respectively as

5 $$B_{{\text{sj}},{\text{p}}} = \frac{{1}}{{2}}\frac{\Phi }{{A_{{\text{sj}}} }},$$

6 $$B_{{\text{rj}},{\text{p}}} = \frac{{1}}{{2}}\frac{\Phi }{{A_{{\text{rj}}} }},$$

where $A_{{\text{sj}}}$ and $A_{{\text{rj}}}$ are the respective flux areas of stator and rotor yoke parts, $A_{{\text{sj}}} \,=\,h^{\prime}_{{\text{sj}}} l_{\text{s}}$ and $A_{{\text{rj}}} \,=\,h^{\prime}_{{\text{rj}}} l_{\text{r}}$. Where $h^{\prime}_{{\text{sj}}}$ and $h^{\prime}_{{\text{rj}}}$ are the respective calculation heights of stator and rotor yokes. They are commonly determined by their geometrical yoke heights together with slot types.

### Loss model under sinusoidal flux excitation

A classical model is the Bertotti’s three-term loss separation model^17^, where the total losses $P$ are divided into three components: the hysteresis loss $P_{\text{h}}$, the classical eddy current loss $P_{{\text{ec}}}$, and the excess loss $P_{\text{e}}$.

7 $$P = P_{\text{h}} + P_{{\text{ec}}} + P_{\text{e}} .$$

If the term of the excess loss in (7) is ignored, then (7) can be reduced to

8 $$P = P_{\text{h}} + P_{{\text{ec}}} .$$

The ignored term of the excess loss is actually included in the other two terms: the hysteresis loss and the classical eddy current loss^15,16^. The unit of the results calculated from (8) is expressed in W/kg.

### Loss model with inverter supply

The Boglietti’s model is a representative loss model of soft magnetic materials (SMMs) with inverter supply^15,16^. The model is derived from (8). In the model, minor hysteresis loops are usually neglected because of high inverter switching frequency. In addition, the additional harmonic eddy current losses due to inverter supply are considered by a model correction factor (MCF)$\chi$. The model can be expressed by a formula as

9 $$P_{{\text{PWM}}} = \overbrace {{\underbrace {{afB_{\text{p}}^{x} }}_{{P_{\text{h}} }} + \underbrace {{bf^{2} B_{\text{p}}^{2} }}_{{P_{{\text{ec}}} }}}}^{{\text{Total fundamental losses}}} + \underbrace {{\left( {\chi^{\text{2}} - \text{1}} \right)\overbrace {{bf^{2} B_{\text{p}}^{2} }}^{{P_{{\text{ec}}} }}}}_{{\text{Total harmonic losses}}},$$

where $a$ and $b$ are the respective hysteresis and classical eddy current loss coefficients, $f$ is a magnetic field frequency, $B_{\text{p}}$ is the peak value of the magnetic flux density, $x$ is the Steinmetz coefficient, $\chi$($\chi \ge \text{1}$) denotes the ratio between the root mean square value of the total voltage from inverter supply and that of its fundamental voltage. On the right side of (9), the sum of the first two terms stands for the total fundamental losses and the last term represents the total harmonic losses. The classical eddy current loss coefficient $b$ is formulated as

10 $$b=\,\frac{{\sigma\uppi ^{\text{2}} d^{\text{2}} }}{{\text{6}\rho }},$$

where $\sigma$, $d$ and $\rho$ are the electrical conductivity, the thickness, and the density of SMMs.

### Calculation of iron losses in spindle motors

The equivalent magnetic circuit (EMC) is combined with (9) to estimate iron losses in the spindle motor. The spindle motor is supplied with a voltage source inverter. The EMC is used to calculate the magnetic flux densities of the spindle motor cores. The loss densities of the spindle motor cores are calculated by (9). The total iron losses in the spindle motor can be calculated from the following formula

11 $$P_{{\text{ir}}} = \overbrace {{K_{\text{1}} m_{{\text{st}}} P_{{\text{PWM}}} \left( {B_{{\text{st,p}}} \text{,}f} \right){ + }K_{\text{2}} m_{{\text{sj}}} P_{{\text{PWM}}} \left( {B_{{\text{sj,p}}} \text{,}f} \right)}}^{{\text{Iron losses in the stator core}}} + \underbrace {{K_{\text{1}} m_{{\text{rt}}} \left( {\chi^{\text{2}} - \text{1}} \right)P_{{\text{ec}}} \left( {B_{{\text{rt,p}}} \text{,}f} \right){ + }K_{\text{2}} m_{{\text{rj}}} \left( {\chi^{\text{2}} - \text{1}} \right)P_{{\text{ec}}} \left( {B_{{\text{rj,p}}} \text{,}f} \right)}}_{{\text{Iron losses in the rotor core}}},$$

where $K_{\text{1}}$ and $K_{\text{2}}$ are the empirical coefficients to consider rotor rotating motion combined with tooth and slot effect, $P_{{\text{PWM}}} \left( {B_{{\text{st,p}}} \text{,}f} \right)$ and $P_{{\text{PWM}}} \left( {B_{{\text{sj,p}}} \text{,}f} \right)$ are the loss densities of the tooth and yoke parts of the stator core, $\left( {\chi^{\text{2}} - \text{1}} \right)P_{{\text{ec}}} \left( {B_{{\text{rt,p}}} \text{,}f} \right)$ and $\left( {\chi^{\text{2}} - \text{1}} \right)P_{{\text{ec}}} \left( {B_{{\text{rj,p}}} \text{,}f} \right)$ are the loss densities of the tooth and yoke parts of the rotor core, $m_{{\text{st}}}$, $m_{{\text{rt}}}$, $m_{{\text{sj}}}$, and $m_{{\text{rj}}}$ are the total masses of the tooth and yoke parts of stator and rotor cores, where $m_{{\text{st}}} \,=\,\rho b_{{\text{st}}} h^{\prime}_{{\text{st}}} Z_{\text{1}} l_{\text{s}}$, $m_{{\text{rt}}} \,=\,\rho b_{{\text{rt}}} h^{\prime}_{{\text{rt}}} Z_{\text{2}} l_{\text{r}}$, $m_{{\text{sj}}} \,=\,\rho\uppi \left( {D_{\text{1}} - h^{\prime}_{{\text{sj}}} } \right)h^{\prime}_{{\text{sj}}} l_{\text{s}}$, and $m_{{\text{rj}}} \,=\,\rho\uppi \left( {D_{{\text{i2}}} + h^{\prime}_{{\text{rj}}} } \right)h^{\prime}_{{\text{rj}}} l_{\text{r}}$. Where $h^{\prime}_{{\text{st}}}$ and $h^{\prime}_{{\text{rt}}}$ are the stator and rotor tooth calculation lengths used for magnetic circuit calculating, and they are related to slot types but the notch heights of slots are usually ignored in them, $D_{\text{1}}$ and $D_{{\text{i2}}}$ denote the outer and inner diameters of stator and rotor cores. In (11), both fundamental and harmonic components from inverter supply are significant contributors to iron losses in the stator core. However, there is almost no contribution of the fundamental component from inverter supply to iron losses in the rotor core owing to a very small rotor slip. The iron losses in the rotor core are mainly caused by the harmonic components from inverter supply. These may be why the loss densities of stator and rotor cores are different.

**Figure 2:**
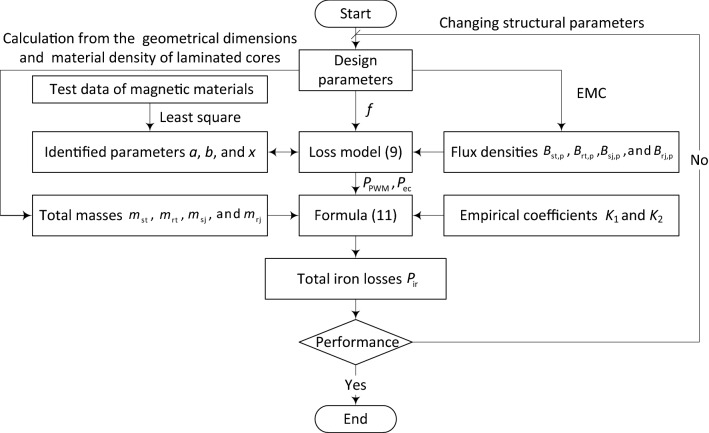
Calculation of iron losses in the spindle motor.

The proposed calculation method of iron losses in the spindle motor is integrated into a developed program^35^ by compiling source codes. The detailed calculation processes of the developed program are shown in Fig. 2.

*Step 1* The data of the spindle motor design parameters, e.g., voltage, frequency, shaft power, the number of pole pairs, and structure and the corresponding geometrical dimensions are input to the developed program before its run.

*Step 2* The magnetic flux density levels of airgap, stator and rotor cores can be calculated using the EMC and the known values of the design parameters.

*Step 3* The loss densities of the tooth and yoke parts of stator and rotor cores can be calculated by (9), the calculation results from Step 2, and a known inverter supply frequency. The loss coefficients of (9) need to be determined from the physical characteristic and experimental data of electrical steel sheets (ESSs) before model use.

*Step 4* The total masses of the tooth and yoke parts of stator and rotor cores can be calculated according to their structure, and the corresponding geometrical dimensions and material densities.

*Step 5* The total iron losses in the spindle motor can be calculated according to (11), and the calculation results from Steps 3 and 4. In fact, they are divided into two parts: static and dynamic iron losses. The static iron losses are produced due to magnetic field alternating. The dynamic iron losses are caused by rotor rotating motion combined with tooth and slot effect. Based on the static iron losses, the dynamic iron losses are commonly considered by introducing empirical coefficients (whose values are always more than 1) because of their complex generation mechanisms.

*Step 6* The calculation will be not stopped until the performance requirements of the spindle motor are satisfied. The performance of the spindle motor can be optimized by changing electromagnetic structure parameters.

### Application to a study case

The studied spindle motor is a three-phase inverter-fed induction motor. The performance parameter values of the spindle motor are calculated and obtained from the developed program^35^, as seen in Table 1.

**Table 1:** Specifications of the spindle motor.

Parameters	Values
Number of pole pairs *p*	2
Rated voltage $U_{\text{N}}$ (V)	380
Rated power $P_{\text{N}}$ (kW)	35
Rated synchronous speed $n_{\text{N}}$ (r/min)	6000
Rated current $I_{\text{N}}$ (A)	67
Rated efficiency $\eta_{\text{N}}$	86.4%
Rated power factor $\text{cos}\varphi_{\text{N}}$	0.92
Number of stator and rotor slots $Z_{\text{1}} /Z_{\text{2}}$	48/38
Stator outer diameter $D_{\text{1}}$ (mm)	160
Stator inner diameter $D_{{\text{i1}}}$ (mm)	110
Rotor outer diameter $D_{\text{2}}$ (mm)	109.1
Rotor inner diameter $D_{{\text{i2}}}$ (mm)	78
Laminated stator and rotor core lengths $l_{\text{1}} /l_{\text{2}}$ (mm)	230/230
Air gap length $\delta$ (mm)	0.45
Stator resistance per phase(@115 °C)$R_{\text{s}}$($\Omega$)	0.1 077
Rotor resistance per phase (@115 °C)$R_{\text{r}}$($\Omega$)	0.1 273
Stator leakage reactance per phase $X_{\text{s}}$($\Omega$)	0.3 940
Rotor leakage reactance per phase $X_{\text{r}}$($\Omega$)	0.4 302
Excitation reactance per phase $X_{\text{m}}$($\Omega$)	19.0 967

**Figure 3:**
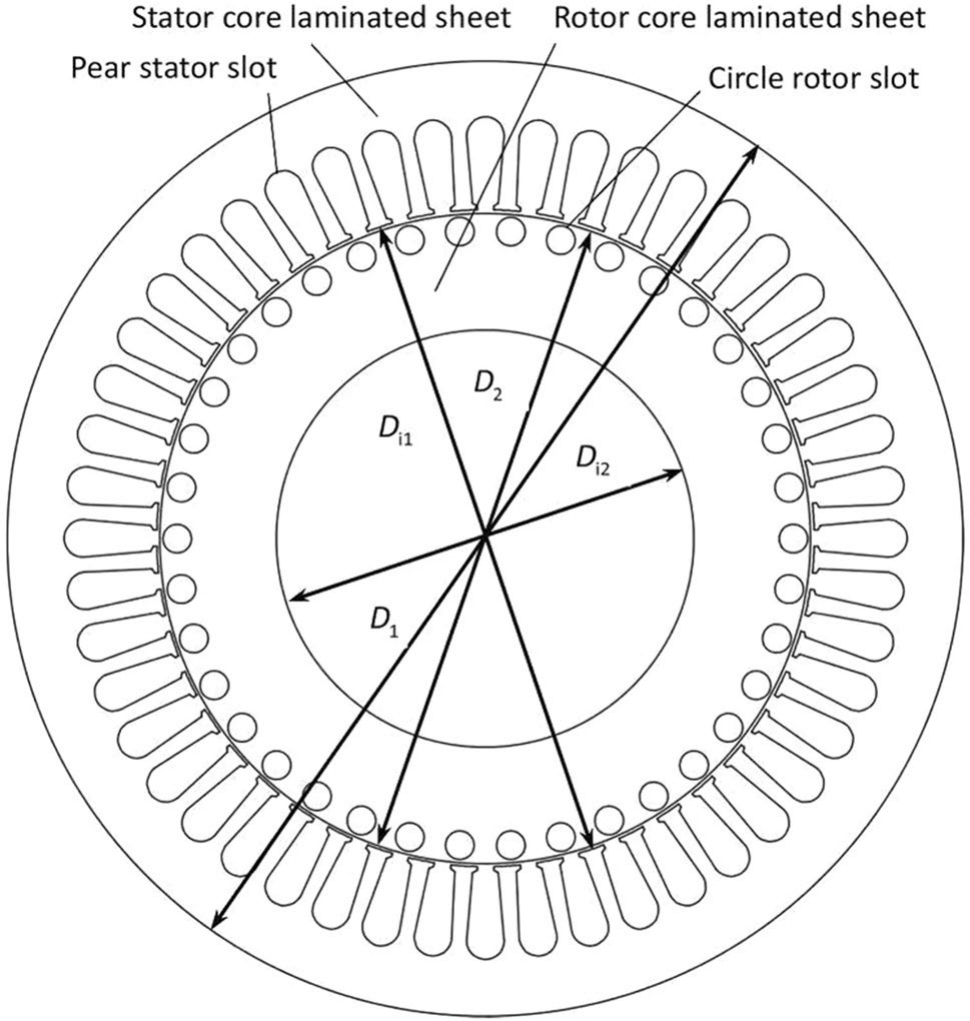
Laminated sheets of stator and rotor cores.

Figure 3 shows the laminated sheets of the stator and rotor cores of the spindle motor. The stator and rotor core laminated sheets have 48 and 38 slots. They are designed respectively as pear and circle ones. All the slots are uniformly distributed in their own circumferential directions. A slot number combination of 48 and 38 is chosen as a final design solution one. In this combination, there is an obvious difference between stator and rotor slot numbers to reduce electromagnetic vibration and noise. The slot number of the stator core laminated sheet is designed as 48. It belongs to a more slot design to obtain a smaller stator outer diameter and more compact structure, and to suppress slot harmonic losses and asynchronous additional torque.

**Figure 4:**
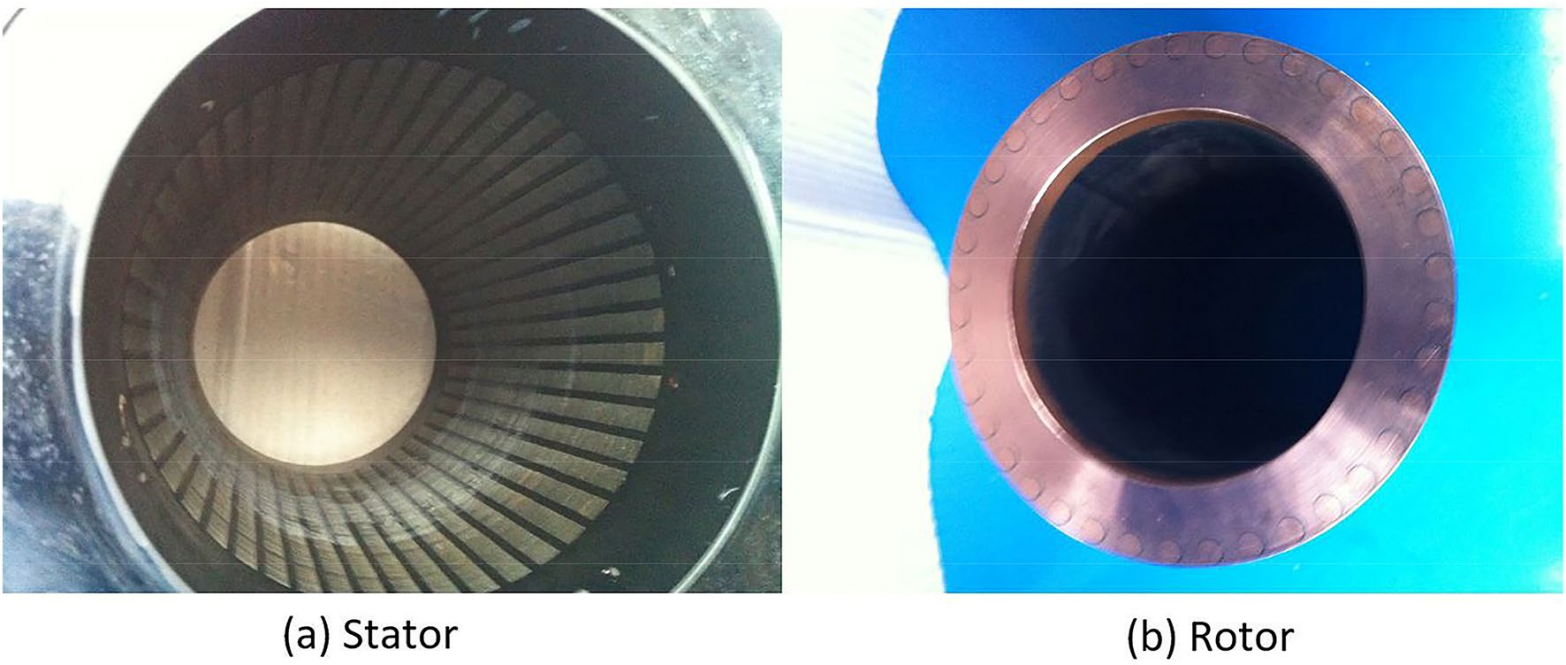
Assembled stator and rotor.

The bars in a rotor cage of the spindle motor are designed as copper ones to reduce rotor resistance, heat generation and slip, and to improve performance (Fig. 4b). 35W300 ESSs are chosen to manufacture the stator and rotor cores of the spindle motor to suppress eddy currents and reduce magnetizing current (Fig. 4) because they have thin thicknesses and high permeability. The properties of 35W300ESSs are given in Table 2.

**Table 2:** Physical and magnetic properties of 35W300 ESSs.

Parameters	Values
Thickness *d* (mm)	0.35
Density $\rho$ (kg/m^3^)	7650
Electrical conductivity $\sigma$($\Omega^{ - 1} {\text{ m}}^{ - 1}$)	3.75 × 10^6^
Specific loss @ 50 Hz and 1.6 T (W/kg)	3.00
Minimum magnetic density @ 2500 A/m (T)	1.55
Minimum magnetic density @ 5000 A/m (T)	1.65
Minimum magnetic density @ 9800 A/m (T)	1.76

### Model parameter extraction

The fundamental parameters in (9) are composed of the hysteresis coefficient *a*, the Steinmetz coefficient *x*, and the classical eddy current coefficient *b*. They can be extracted from the physical characteristic and experimental data of 35W300 ESSs before model use. In the case of sinusoidal flux waveform excitation, the value of the parameter $\chi$ in (9) is equal to 1. The Steinmetz coefficient *x* is assumed as a constant value of 2 because the desired results of iron losses in motors can be achieved^36,37^. The value of the parameter *b* is calculated and obtained from (10) and the known physical characteristic data of 35W300 ESSs (Table 2). Based on (9), the measured data of 35W300 ESSs under 50 Hz sinusoidal excitation are fitted by the least square to obtain the value of the parameter *a*. As a result, the values of the fundamental parameters in (9) are given in Table 3.

**Table 3:** Model parameters and the corresponding values.

Parameters	Values
Hysteresis coefficient *a* ($\text{W} \, \text{kg}^{{{ - 1}}} \, \text{Hz}^{{{ - 1}}} \, \text{T}^{{{ - 2}}}$)	1.78 × 10^–2^
Steinmetz coefficient *x*	2
Eddy current coefficient *b* (${\text{W kg}}^{ - 1} {\text{ Hz}}^{ - 2} {\text{ T}}^{ - 2}$)	9.88 × 10^–5^

### Experimental verification

**Figure 5:**
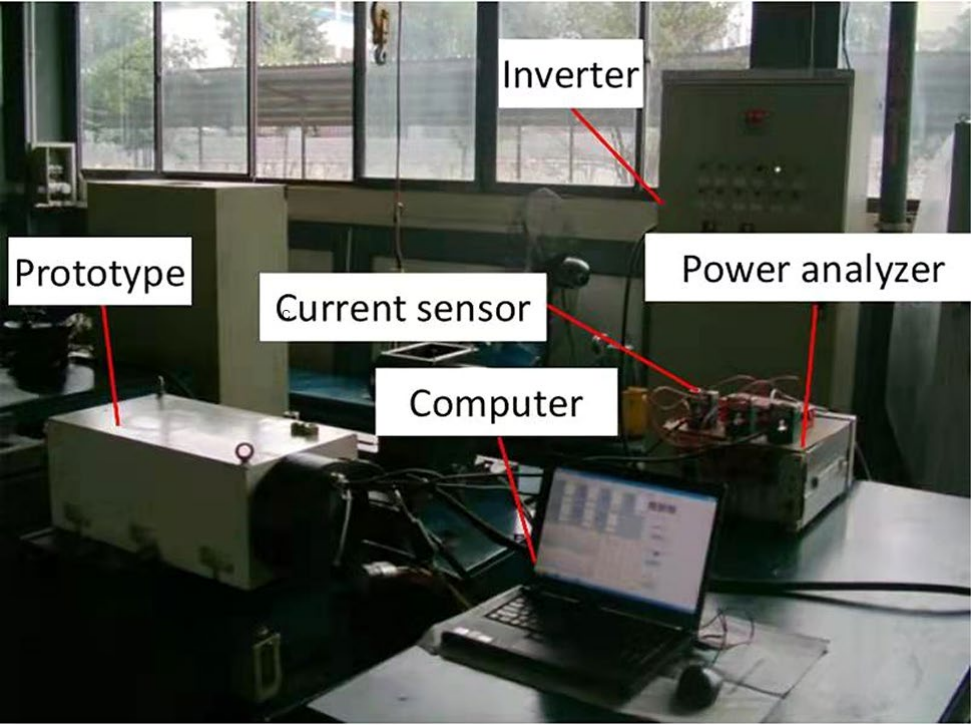
Prototype testing.

The experiment is performed on a developed and manufactured motor spindle prototype (Fig. 5) which is supported by hydrostatic bearings. The rated power and rotational speed of the prototype are 35 kW and 6 k r/min, respectively. The specifications of the prototype motor are exactly the same as those of the spindle motor in Table 1. The prototype works under inverter supply.

#### Mechanical loss separation and measurement using a novel method

The mechanical loss is a major loss of the prototype. It is very important to correctly separate the mechanical loss component of the prototype from its total losses by experiment. Proper separation methods can ensure the accuracy of experimental results.

The mechanical loss of motor spindles is usually separated and measured using the existing method^38^. In the method, two exactly same motor spindles are used and connected to each other by a coupling device, and either of them can be used as a drive or load motor spindle. At a given speed, the mechanical loss of motorized spindles can be obtained by measuring the difference between the input active power of the drive motor spindle without and with load, but the load motor spindle is always powered off during a whole testing process. In this section, a novel and simpler method is proposed. It is a combination of no load running and a sudden power supply cut. In the proposed method, only one motor spindle is needed during a whole testing process. There is no need for any other same motor spindle and a coupling device.

The no load running is combined with a sudden power supply cut to separate the mechanical loss of the prototype from its total losses. The inverter-fed prototype is started under no load. Then the speed of the prototype rises to a rated speed of 6 k r/min by changing inverter supply frequency. Under no load, the prototype is kept running at the rated speed for long enough time so that its temperature rise is not changed any more. When the temperature rise is stable, the power supply of the prototype is suddenly cut off to eliminate its braking torque and electromagnetic losses. At this moment, only the mechanical loss is left. After suddenly losing power supply, the speed of the prototype will not decrease to zero immediately but show a drop process due to bearing and rotor wind forces of friction.

The mechanical loss of the prototype is assumed as a function of rotational speed square

12 $$P_{\text{m}} \,=\,c\omega_{\text{m}}^{{2}},$$

where $P_{\text{m}}$ denotes the mechanical loss of the prototype running at any speed, $c$ is the rotor rotating friction factor, and $\omega_{\text{m}}$ is the mechanical angular velocity of the prototype rotor. The mechanical loss of the prototype can be measured by measuring the friction factor $c$ and the rotational speed.

According to the conservation law of energy, under suddenly losing power supply, the loss of kinetic energy of the prototype in any time interval is exactly equal to the work done to overcome frictional forces in the same time interval. The conservation equation of energy is written as

13 $$- \int_{{t_{{1}} }}^{{t_{{2}} }} {P_{\text{m}} \text{d}t} =\,E_{{\text{k2}}} - E_{{\text{k1}}}=\,\frac{{1}}{{2}}J\left( {\omega_{{\text{m2}}}^{\text{2}} - \omega_{{\text{m1}}}^{\text{2}} } \right),$$

where $t_{\text{1}}$ and $t_{\text{2}}$ denote two different rotating motion moments of the prototype after a sudden power supply cut and they can be measured by a stopwatch, $E_{{\text{k1}}}$ and $E_{{\text{k2}}}$ are the kinetic energy of the prototype at the moments $t_{\text{1}}$ and $t_{\text{2}}$, $J$ is the total moment of inertial of the prototype including a motor rotor, and a shaft and its accessories, which can be calculated from rotor structure, and the corresponding geometrical dimensions and material densities ($J = 0.0 583\, {\rm kg\, m}^{2}$), $\omega_{{\text{m1}}}$ and $\omega_{{\text{m2}}}$ are the measured mechanical angular velocities of the prototype rotor at the moments $t_{\text{1}}$ and $t_{\text{2}}$ by a rotational speed meter.

After a sudden loss of power supply, the mechanical angular velocity of the prototype rotor is assumed as a polynomial

14 $$\omega_{\text{m}} \left( t \right)=\,c_{\text{0}} t^{\text{3}} { + }c_{\text{1}} t^{\text{2}} { + }c_{\text{2}} t{ + }c_{\text{3}} ,$$

where $t$ denotes any moment of the prototype after a sudden power supply cut, and $c_{\text{0}}$, $c_{\text{1}}$, $c_{\text{2}}$ and $c_{\text{3}}$ are the polynomial coefficients.

The measured data of a speed change in the prototype after a sudden power supply cut are listed in Table 4.

**Table 4:** Measured results of a speed change in the prototype.

Speed 1 at *t*_1_ = 0 s (k r/min)	Speed 2 (k r/min)	Av. time required from speed 1 to speed 2 *t*_2_ (s)
6	5	1.35
6	3	5.08
6	1	12.50

Based on the measured data in Table 4, (14) is combined with (12) and (13) to solve the value of the friction factor $c = 0.0 080$ $\text{N m s rad}^{{ - {1}}}$.

#### Iron loss test

A simpler method is used to measure iron losses in the prototype than the traditional method. In the method, there is no need for connecting the prototype under test to a synchronous motor with the same pole pairs. The no load active power of the prototype rotating at different speeds is measured by a power analyzer. Actually, under no load, the current size of the prototype running at any speed exceeds a power analyzer current limit value of 5 A. Current sensors are required in the test. The current sensors with a transformer ratio of 1:40 can be chosen from a power capacity of the prototype.

The iron losses in the prototype can be indirectly obtained from the following formula

15 $$P_{{\text{ir}}} = P_{{\text{no-load}}} - P_{\text{m}} - mR_{\text{s}} I_{\text{s}}^{\text{2}} ,$$

where $P_{{\text{ir}}}$ denotes the total iron losses, $P_{{\text{no-load}}}$ is the measured no load active power, $P_{\text{m}}$ is the measured mechanical loss, $m$ is the number of phase, $R_{\text{s}}$ and $I_{\text{s}}^{{}}$ are the measured respective stator winding resistance and current per phase.

In (15), both stray and rotor resistance loss terms are neglected, but they are included in the total iron losses. The term of the stray loss at no load is very small so that it can be ignored. The term of the rotor resistance loss can also be ignored because of a very low rotor slip at no load. The good measurement accuracy can be still guaranteed even without considering them.

## Results and discussion

**Figure 6:**
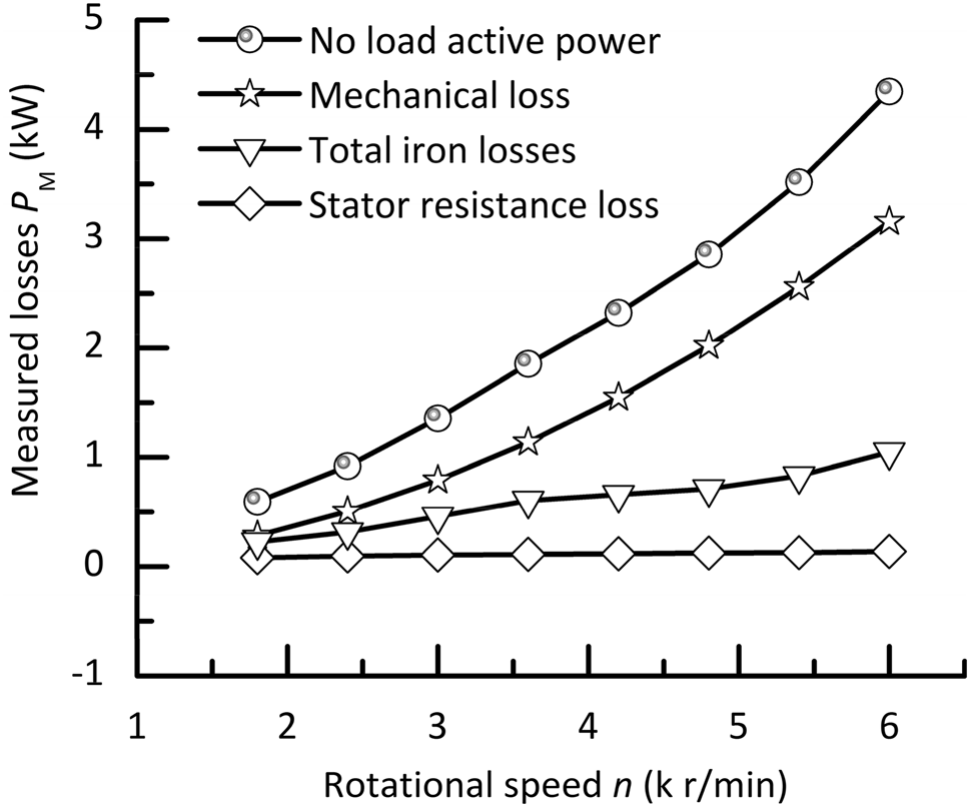
Experimental curves of various losses of the prototype under no load.

Figure 6 shows the measured results of various losses of the no load running prototype at different speeds. They include the stator resistance loss, the mechanical loss, the iron loss, and the total losses (input active power). Within a rated speed range, the stator resistance loss of the prototype is almost not changed in spite of speed changing, but the other losses of the prototype vary at different speeds.

Within a rated speed range, the stator resistance loss of the no load running prototype is not sensitive to its speed change. In this speed region, a constant *V/f* or magnetic flux or torque control strategy is used for the prototype. In the control method, no matter how the speed of the prototype is changed, while the inactive power exciting or magnetizing current component of the prototype is always kept unchanged. In addition, the input voltage of the prototype increases proportionally as its speed increases. This makes the change in the active power current component of the prototype small. As a result, within a rated speed, despite the speed change, the total current of the no load running prototype is almost constant, and the corresponding stator resistance loss is also almost constant.

The mechanical loss of the prototype is caused by bearing and rotor wind forces of friction. It is proportional to speed square. A higher speed leads to a bigger mechanical loss value of the prototype.

Within a rated speed range, the total iron losses in the prototype increase with an increase in its rotational speed. The reasons may be summarized into two aspects. First, within a rated speed range, the magnetic flux of the prototype is kept unchanged because of adopting a *V/f* control strategy. However, with a higher rotational speed or magnetic field frequency, it results in the increase of the static iron losses in the prototype due to magnetic field alternating. Second, the dynamic iron losses in the prototype are increased with the rotational speed because of rotor rotating motion combined with tooth and slot effect.

At a rated speed without load, the stator resistance, mechanical, and iron loss terms of the prototype account for about 0.4%, 9%, and 3% of its rated power (35 kW), respectively. The mechanical loss is a major loss component of the prototype. In a prototype test, a proper mechanical loss separation method is very important for obtaining accurate experimental results of iron losses.

The prototype is supported by hydrostatic bearings. The bearing is a main contributor to the mechanical loss of the prototype. Also it is a main heat source. In the development and design of this type of motorized spindles, to obtain lower temperature rise and better comprehensive performance, the structure optimization is needed. On the one hand, the generation heat of the bearing and motor of motorized spindles can be reduced as much as possible. On the other hand, in unit time, more heat can be removed from motorized spindles.

**Figure 7:**
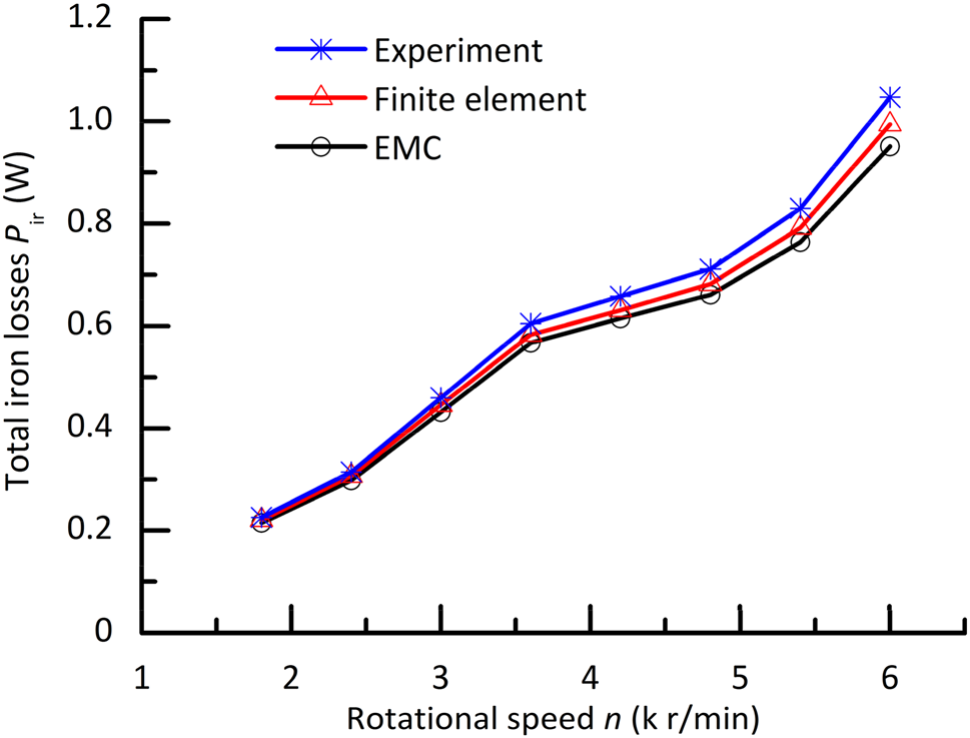
Comparison between the estimated and measured results of iron losses in the prototype at no load.

Figure 7 shows a comparison between the theoretical and experimental curves of iron losses in the prototype under no load. It can be known from Fig. 7 that the calculation accuracy of the finite element is better than that of the EMC. The biggest error between the analysis results of the finite element and the experimental ones is about 5%. Contrasted to the finite element, the analysis results of the EMC are more deviated from the experimental values. In the EMC, the magnetic flux density distribution of any section inside motors is assumed as a uniform one. This leads to a decrease in the calculation accuracy of the method. However, the analysis results of the EMC still agree well with the prototype experiment data. The error between the estimated and measured results is no more than 10%. The validity of the proposed method is confirmed by experiment and a different analysis method comparison. In the proposed method, there is no need to solve a complex electromagnetic field inside motors, and to perform 2D or 3D eddy current analysis and the corresponding post-processing. It needs to only perform a simple magnetic circuit calculation. Therefore, it can achieve a fast analysis and prediction.

## Conclusion

The equivalent magnetic circuit (EMC) method is proposed to estimate iron losses in the spindle motor. In the method, the magnetic flux density distribution of any cross section inside the spindle motor is assumed as a uniform one. The EMC is used along with the Boglietti’s model. They are integrated into a developed program by compiling source codes to achieve an analysis. A mechanical loss separation method of no load running combined with a sudden loss of power supply is also proposed to eliminate the braking torque and electromagnetic losses of the spindle motor. The analysis results of the EMC and the finite element are compared with the experimental ones. The calculation errors of two methods are analyzed and discussed. It is demonstrated that the error between the results calculated from the EMC and the measured values is no more than 10%. The validity of the proposed method is proved by experiment and a different analysis method comparison.
